# Comparable Low-Level Mosaicism in Affected and Non Affected Tissue of a Complex CDH Patient

**DOI:** 10.1371/journal.pone.0015348

**Published:** 2010-12-21

**Authors:** Danielle Veenma, Niels Beurskens, Hannie Douben, Bert Eussen, Petra Noomen, Lutgarde Govaerts, Els Grijseels, Maarten Lequin, Ronald de Krijger, Dick Tibboel, Annelies de Klein, Dian Van Opstal

**Affiliations:** 1 Department of Pediatric Surgery, Erasmus-MC Sophia, Rotterdam, The Netherlands; 2 Department of Clinical Genetics, Erasmus-MC Sophia, Rotterdam, The Netherlands; 3 Department of Obstetrics and Gynaecology, Erasmus-MC Sophia, Rotterdam, The Netherlands; 4 Department of Radiology, Erasmus-MC Sophia, Rotterdam, The Netherlands; 5 Department of Pathology, Erasmus-MC Sophia, Rotterdam, The Netherlands; University of Giessen Lung Center, Germany

## Abstract

In this paper we present the detailed clinical and cytogenetic analysis of a prenatally detected complex Congenital Diaphragmatic Hernia (CDH) patient with a mosaic unbalanced translocation (5;12). High-resolution whole genome SNP array confirmed a low-level mosaicism (20%) in uncultured cells, underlining the value of array technology for identification studies. Subsequently, targeted Fluorescence In-Situ Hybridization in postmortem collected tissues demonstrated a similar low-level mosaicism, independently of the affected status of the tissue. Thus, a higher incidence of the genetic aberration in affected organs as lung and diaphragm cannot explain the severe phenotype of this complex CDH patient. Comparison with other described chromosome 5p and 12p anomalies indicated that half of the features presented in our patient (including the diaphragm defect) could be attributed to both chromosomal areas. In contrast, a few features such as the palpebral downslant, the broad nasal bridge, the micrognathia, microcephaly, abnormal dermatoglyphics and IUGR better fitted the 5p associated syndromes only. This study underlines the fact that low-level mosaicism can be associated with severe birth defects including CDH. The contribution of mosaicism to human diseases and specifically to congenital anomalies and spontaneous abortions becomes more and more accepted, although its phenotypic consequences are poorly described phenomena leading to counseling issues. Therefore, thorough follow–up of mosaic aberrations such as presented here is indicated in order to provide genetic counselors a more evidence based prediction of fetal prognosis in the future.

## Introduction

### CDH and somatic (chromosomal) mosaicism

Congenital Diaphragmatic Hernia (CDH) is a severe birth defect characterized by defective formation of the diaphragm, lunghypoplasia and pulmonary hypertension. Its overall prevalence is 1/3000 live births and the majority are left sided. Associated anomalies (i.e. non-isolated cases with or without an abnormal karyotype) are involved in 60% of cases. The mortality rate is still high: 10–20% for isolated and up to 40% for non-isolated cases. CDH is increasingly detected by structural ultrasound in the second trimester of pregnancy showing features like a mediastinal cardiac shift away from the side of the defect and an intra-thoracic stomach bubble [1]. From a clinical point of view, early detection enables counseling by a pediatric surgeon and/or clinical geneticist. Parents may opt for a termination of pregnancy and in case of continuation; obstetric and postnatal management can be optimized with referral to a specialized tertiary centre with ECMO (Extra Corporal Membrane Oxygenation) facilities.

The presumed multifactorial etiology of CDH is still poorly understood. Yet, the identification of human chromosomal “hot spots” presents strong evidence for a genetic component [2]–[3]. Because chromosomal aberrations are detected in 10–20% of the cases, regular cytogenetic analysis by GTG-band karyotyping and FISH are highly recommended in each patient [3]. In addition, performance of high-resolution whole genome array could be valuable, especially in case of additional malformations such as in the cardiovascular system, genito-urinal tract and central nervous system [4]. Furthermore, CDH may be part of a defined syndrome, the most well recognized one being Fryns (OMIM 229850 with over 80% of the patients showing CDH, though its locus remains elusive. Finally, CDH caused by tissue-limited mosaicism, specifically of tetrasomy 12p, is rather common within the Pallister-Killian syndrome (PKS)(OMIM 601803 [5]). However, in literature only a few other types of mosaic abnormalities have been described in association with CDH [6]–[10]. Difficulties in detecting (low-level) mosaicism and the former absence of practical, high-resolution whole genome screening techniques could partly account for these low incidences.

Mosaicism is defined as “the presence of a mixture of cells of different genetic composition in a single organism” [11]–[12]. Advances in molecular cytogenetic techniques have allowed for a systematic whole genome screening of genetic aberrations among others in mosaic form. Results indicated that such mosaicism probably exists at a higher frequency than expected [12]–[13]. To date, published studies on this topic both included screens of diseased and (although very few) phenotypically normal individuals and focused on single nucleotide mutations/polymorphisms [14]–[15] as well as structural chromosomal aberrations/variations [16]–[23]. It is accepted that the phenotypic consequences of these somatic mutations depend on the type of cells involved, the nature of the initial mutation and its timing. Somatic mosaicism is also known to produce often a milder phenotype than in its non-mosaic form, allowing for survival of some disorders/aneuploidies that would otherwise result in lethality. Germ-line and somatic mosaicism are thus acknowledged as important factors contributing to phenotypic variability [11]. Nonetheless, in general the significance of somatic mosaicism usually remains under-appreciated and performance of detailed postmortem follow-up of the aberrations in multiple tissues is done only rarely. Consequently, real understanding whether and how chromosomal mosaicism has the potential to mediate tissue-specific dysfunction in affected (and phenotypic normal) individuals largely remains elusive.

In this case report we present the detailed clinical and molecular cytogenetic analysis of a low-mosaic unbalanced translocation (5;12) identified in a complex-CDH patient. The identification of this specific chromosome aberration with high resolution whole genome SNP array on umbilical cord blood shows the value of array technology for identification studies, also in cases of low mosaicism. Furthermore, we hypothesized in the patients' affected tissues a higher contribution of abnormal cells, which was assessed by FISH analysis in several tissues. Finally, results of phenotype-genotype correlations of each characteristic in this patient are presented.

## Materials and Methods

### Routine Cytogenetic and targeted FISH analysis

Ethics approval was provided by the Medical Ethics Committee of the Erasmus Medical Centre, Rotterdam, The Netherlands. A written informed consent was obtained from all participants in this study.

Sixteen ml of amniotic fluid (AF) was cultured using the in situ method in BD Falcon Culture slides (VWR, Amsterdam, The Netherlands) and subsequently used for both GTG-banded karyotyping and fluorescence in situ hybridization (FISH). Karyotyping of the parents was executed on GTG banded metaphase spreads obtained from peripheral blood cultures. Karyograms were reported according the ISCN rules. Fetal skin and umbilical cord tissue were cultured according to standard techniques and used for FISH analysis in a postnatal diagnostic setting.

FISH experiments were performed according to standard protocols with minor modifications and analyzed with an Axioplan 2 Imaging microscope (Zeiss, Sliedrecht, The Netherlands). Images were captured using Isis software (MetaSystems, Altlussheim, Germany). BAC clones were selected from the UCSC genome browser (UC Santa Cruz, Santa Cruz, CA, http://genome.ucsc.edu/cgi-bin/hgGateway, assembly March 2006) and purchased from BACPAC Resources (Oakland,CA). BAC probes (Table S1) were subsequently validated on control metaphases. Targeted whole chromosome paints 5 and 12 and probes of the Cri-du-Chat/Cornelia-de-Lange region were commercially available from Eurodiagnostics (Arnhem, Netherlands) and Cytocell (http://www.cytocell.com/products/aquarius/micro/cri.asp) respectively.

### MLPA

DNA was isolated from 2×2 ml of uncultured AF using the Chemagenic Magnetic Separation Module I (Chemagen, Baesweiler, Germany). MLPA analyses were performed using the SALSA P036 and P070 kits of MRC-Holland (Amsterdam, the Netherlands). These kits contain probes for all subtelomeric p-and-q arm regions of all chromosomes. In addition, a specific aneuploidy sensitive kit (P095) and a mental retardation focused kit harbouring 6 probes in the 5p15 telomeric region were executed. MLPA reactions were performed using the manufacturer's protocol with minor modifications [24] and amplification products were separated and quantified as described previously [25]. Dosage ratio values of <0.7 and >1.3 were used as boundaries for deletions and duplications respectively with use of specific calculated cut-off values in case of kit P095.

### High resolution SNP array

High-resolution whole genome analysis was performed on umbilical-cord blood derived DNA, using the Illumina Quad610 genotyping bead chip (Illumina, San Diego, CA, USA) according to the manufacturer's protocol. This array contains 598 821 SNP probes distributed genome wide and an additional 21 890 intensity-only probes in regions where SNP coverage is poor. Image intensities were extracted using the Illumina's BeadScan software. Data for each BeadChip were self-normalized in Beadstudio GT (version 3.0+) using information contained within the array (http://www.illumina.com/applications). CNVs were detected by comparison to the common Illumina-reference set of 87 CEU samples specific for the Q610 array and visualized in Nexus (version 4.1) software as log2 ratios (http://www.biodiscovery.com/index/nexus). The analysis settings were as follow: SNP rank segmentation with a significance threshold of 1×10-6 and log-ratio-thresholds of 0.2 and −0.2 for duplication and deletion respectively. Mosaic changes were assessed by looking for aberrations in probe intensities along with a shift in genotype frequencies of the SNP probes (measured by b-allele frequencies).

### FISH on paraffin embedded tissue of multiple samplings and placental cryo-sections

Interphase FISH was performed on 4 µm thick paraffin embedded tissue sections. The tissue sections were placed on poly-L-Lysine coated slides. After deparaffinization using xylene, slides were processed in 1 M NaSCN for 10 min at 80°C. Next, pepsin digestion was optimized for each specific tissue type and slides were dehydrated in alcohol. Denaturation of the probes was carried out for 2 min at 95°C. Hybridization was performed at 37°C for 14-16 h. The slides were then washed in posthybridization wash buffers at 55°C and 42°C for 3×5 min and counterstained with DAPI. Signals were counted in at least 100 cells. Results were expressed as the percentage of chromosome 5p signal (RP11-7M4, green) to control probe signal (RP11-90P7, red, Table S1). Internationally accepted cut-off values of >15% of the nuclei with one signal to identify deletions were adapted from the available tumor tissue literature [26].The expected percentage in a normal case is 100%; which means that there is no gene deletion. Placental nuclear suspensions were harvested as described before [27] and fixated in methanol/acetic acid. FISH was performed according to local protocols.

## Results

### Patient report, Cytogenetic analysis and MLPA

A 22-year old Moroccan patient was referred to our tertiary centre for follow up and genetic counseling of a CDH detected in her unborn child by 20-weeks structural ultrasound in her first pregnancy. The medical history was unremarkable. There was no family history of congenital anomalies; however she and her partner were consanguineous in the 2^nd^ line. Ultrasound examination revealed a defect in the left posterolateral diaphragm and herniation of intestine, stomach and liver. Fetal biometry measurements were on the 2^nd^ percentile. Prognosis concerning lung hypoplasia seemed critical with a lung head ratio of <1.0 (0.61 at 21 3/7 weeks) and an intrathoracic liver [28]. Amniocentesis was performed at 20 weeks and 2 days.

Routine cytogenetic analysis confirmed the clinical suspicion of a chromosomal abnormality and revealed a high-mosaic deletion of chromosome 5 in cultured amniocytes which was confirmed by FISH with probes specific for chromosome 5: the karyotype on cultured AF cells was 46,XX,del(5)(p13.3)[27]/46,XX [4] (Table 1 and Table S1). Both parents had a normal karyotype. Based on the paint result an unbalanced translocation was suspected. Therefore MLPA with subtelomeric kits P036 and P070 was performed on DNA isolated from uncultured amniotic fluid cells. Surprisingly, results did not reveal such an anomaly showing normal relative probe signals for all probes including the one on 5p. MLPA using kit P096 which includes 6 probes within the Cri-Du-Chat (CDC) critical region, showed a normal result as well, most probably indicating a much lower level of mosaicism in uncultured AF cells compared to cultured cells.

**Table 1:** Detection of mosaicism using molecular cytogenetic studies.

Detection			
Period	Method	Tissue	Mosaic level (%)
***Prenatal***	GTG-band/FISH	AF-c	87
	MLPA	AF	normal
***Postnatal***	FISH	FUM	43 (38*)
		DER	35 (23*)
	SNP array	UC-bl	20
	FISH Parafin Embedded	Heart	24
		Liver	32
		Diaphragm	26
		Lung	29
		Kidney	28
	FISH Frozen	Placental	13
		DER	36

AF: Amniotic Fluid, AF-c: Amniotic Fluid-cultured.

DER: skin, FUM: Fibroblast culture UMbilical cord.

UC-bl: Umbilical Cord Blood;

*metaphase.

Based on the ultrasound and cytogenetic findings, the parents decided to terminate the pregnancy at 23 1/7 weeks of gestation. A female fetus was delivered vaginally and postmortem physical examination showed a normally proportioned but intra-uterine growth (length) restricted fetus; weight (490 gr: normal: 524+/−116 gr), crown-rump length (17.5 cm; normal 20.8+/−1.9 cm) and foot-length 3.5 cm (normal:4.2+/−0.5 cm). Dysmorphological features were significant for the following: convex eye globes, abnormal palpebral (down) slant, hypertelorism, wide nasal bridge, flat philtrum, thin upperlip, micro-and-retrognathia and nuchal wideness. Abnormal external ear development on both sides and aberrant posterior rotation of the ears were noted as well. Concerning the extremities, a palmar crease on the right was seen and also an abnormal positioning of the anus with short perineum and sacral dimple. Inspection of the internal organs presented a large left diaphragmatic defect of the Bochdalek type with herniation of stomach, duodenum and parts of colon, liver and spleen. Further, this fetus showed severe lung hypoplasia (right 2.1gr, left 1.2 gr; lung-body ratio <<0.015) and left kidney-agenesis with compensatory right kidney hyperplasia. Finally, autopsy demonstrated normal morphology of all remaining organs and no structural aberrations of the brain on postmortem MRI.

The prenatally identified mosaicism 46,XX,del(5)(p13.3) in cultured amniotic fluid cells was confirmed by postmortem cytogenetic analysis in umbilical cord and skin tissue, yet in a much lower frequency than expected (Table 1). Because the suspicion of an unbalanced translocation persisted, yet not confirmed by MLPA on uncultured cells, a whole genome SNP array was applied.

### High-Resolution SNP-array

**Figure 1:**
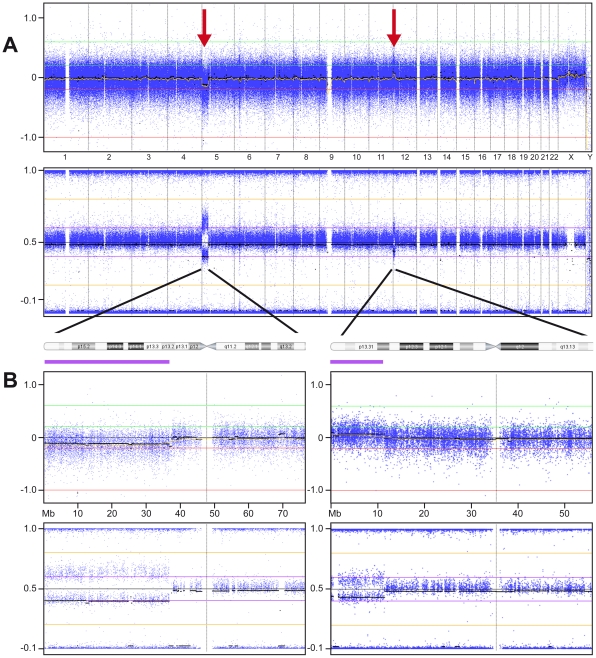
Results High-resolution SNP array using uncultured umbilical cord blood material. A: Whole genome array: Log2 intensity (Top) and B-Allele Frequencies BAF (Bottom). Top: Y-axis: Relative Copy Number State. X-axis: autosomes number 1–22 and the two sex chromosomes. Displayed are the relative and normalized log2 intensities of all available SNPs on the array across the patients' genome. At the beginning of chromosome 5 a slight drop of log2 intensity is visible representing the deletion of part of the short arm of chromosome 5 in a low-mosaic state. Similarly, the mosaic 12p duplication is depicted as a small peak of log2 intensity at the beginning of chromosome 12. Bottom: confirmation of aberrations by the more clearly visible changes in BAF. B: Enlarged views of the results of chromosome 5 (left) and 12 (right) showing both the log2 intensity window as well as the BAF results.

**Figure 2:**
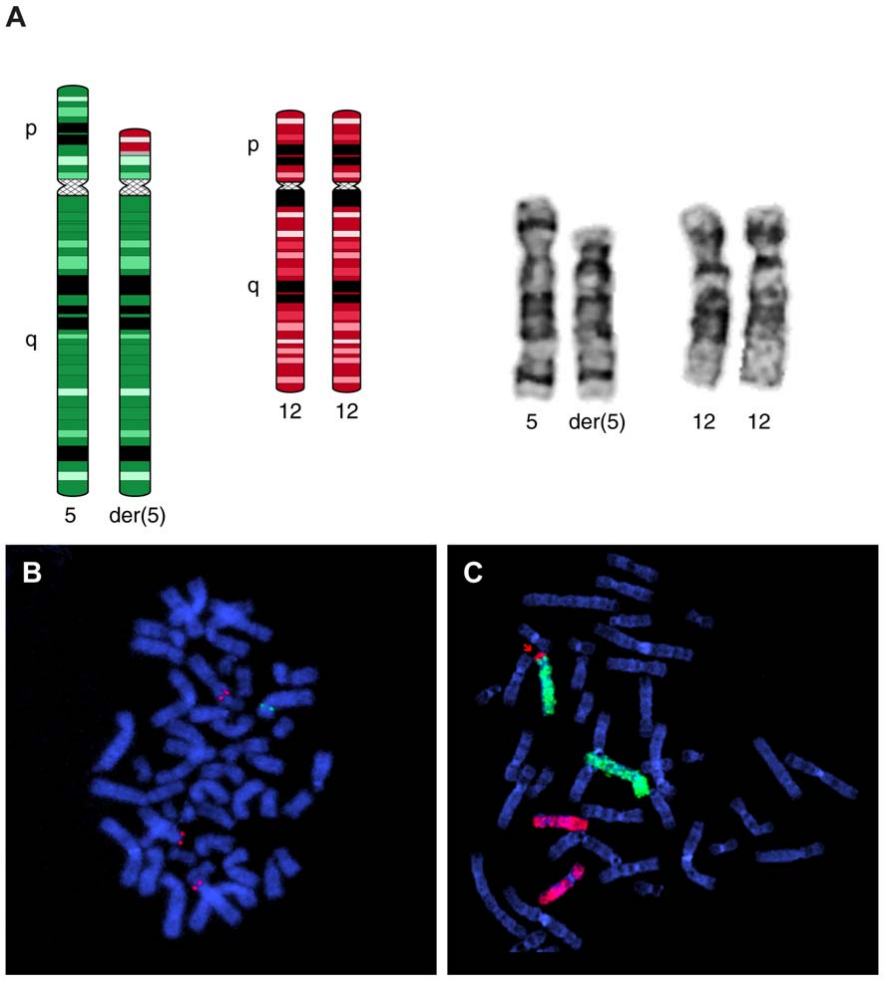
Fluorescent In-Situ Hybridization results using cultured amniotic fluid and skin material. A: Left: Schematic representation of the unbalanced der(5)t(5;12)(p13.2;p12.3) and its normal chromosome 12 counterpart. Right: Partial Karyotype of the patient showing both the normal and abnormal chromosome 5 and both normal chromosomes 12. B: Fluorescent In-Situ Hybridization (FISH) on a metaphase spread of 1 umbilical cord-fibroblast-cell presenting 1 copy of BAC clone RP11-7M4 (Green) on the normal chromosome 5 and 3 of clone RP11-62P15 (red) on both chromosomes 12 and the der(5) C: Whole-Chromosome Paint FISH results of 1 AF-cell showing duplication of part of the short arm of chromosome 12 (WCP12: red) at the expense of part of 1 copy of chromosome 5 (WCP5:green).

High-resolution whole genome SNP array on DNA from umbilical cord blood confirmed the 5p deletion and indeed identified a coexisting chromosome 12p13.2 duplication by using information of both log2 intensities and B-allele frequency as described recently (Figure 1) [29]. These array results were subsequently confirmed by FISH using chromosome 12 paint in addition to a 12p13.32 probe on cultured AF (Figure 2). So, ultimately the patients karyotype could be summarized as 46,XX,der(5)t(5;12)(p13.2;p12.3).arr 12p12.3(rs7136701- rs10845353)x3,5p13.2(rs1108867-rs16903304)x1 (NCBI Build 36/hg18 [30]). Considering the mosaic rate, B-allele ratios of the array suggested a different level of mosaicism in the umbilical cord blood as present in the cultured AF cells. The estimated percentage of mosaicism based on these array results was 20%.

### FISH on different paraffin embedded tissues and cryosections of placenta

Considering the big difference between mosaic rates of cultured amniocytes and umbilical cord blood as well as uncultured AF-cells, we hypothesized that the genetic aberration rate could be high in affected tissues (such as lungs, kidneys and diaphragm) and lower in normal ones (heart, liver). Therefore we screened 7 different paraffin embedded tissues sections with FISH using specific BAC clones. Results are summarized in Table 1; showing no significant differences however in the levels of mosaicism between normal and affected tissue. The derivative chromosome 5 was detected in 24–36% of the cells overall, confirming the array-based estimated percentage of mosaicism. Finally, to assess a co-existing placental mosaicism we also screened frozen sections of placental tissue after nuclear isolation revealing mosaicism in 10% of the nuclei.

### Genotype-Phenotype comparison

Structural aberrations of chromosome region 5p (clinically recognized as the Cri-du-Chat syndrome or as the Cornelia de Lange syndrome) and 12p (featuring as PK syndrome or a pure mosaic trisomy 12p) are both associated with CDH. Yet, for chromosome 5p diaphragmatic hernia has only been associated with Brachmann-de-Lange patients and not with those featuring Cri-du-Chat. Subsequently, the diaphragmatic candidate region on 5p most likely overlaps with the critical region of CdLS, taking into account that CdLS patients are genetically heterogenous as well. Hulinksy et al [31] identified a child with both CDH and CdLS which had a pure interstitial deletion of 5p [del(5)(p13.1;p14.2)]. Results of the comparison with this patient, and for all other characteristics in our patient compared to those described in literature (for both regions) [32]–[33] [34]–[35] [36]–[37] are summarized in Table S2. Based on these comparisons half of the features present in our patient could be attributed to both chromosomal areas. Exceptions to this were: the palpebral downslant, the broad-nasal bridge, the retro-and micro-gnathia, microcephaly, abnormal dermatoglyphics and IUGR, which better fit the 5p associated syndromes.

## Discussion

In this case report we present the detailed clinical and cytogenetical analysis of a unique low-mosaic der (5) t(5;12) aberration identified in a CDH patient with multiple congenital anomalies. Classification of the true chromosome abnormality was possible only after performance of a high-resolution whole genome SNP array on uncultured umbilical cord blood. Application of this technology in the identification of mosaic aberrations was reviewed very recently [29]. Although mosaic abnormalities are increasingly considered to be important factors in (spontaneous) abortions [38], congenital anomalies [22], [39]–[42] and in phenotypic variability, analysis of multiple tissues in prenatal cases is done only rarely. In addition, data on chromosomal mosaicism frequencies in phenotypically normal individuals, with the exception of studies in reproductive tissues [13], is sparse as well [11], [21]. While the clinical significance of the aberrant cell line was quite obvious in our patient due to the presence of major associated birth defects, this is not always the case. Therefore, the recent availability of more sensitive and easier applicable techniques will lead to increased detection of low-mosaic aberrations at a prenatal stage and subsequently lead to more genetic counseling problems. Extensive follow–up of mosaic aberrations in affected cohorts as well as research of the incidence in the normal population is indicated in order to provide genetic counselors a more evidence based prediction of fetal prognosis in the future.

### Phenotypic variability at the tissue level correlated to mosaic frequency?

Because the mosaic aberration was present in both placenta- and embryonic material we suggest that the translocation (and subsequent loss of the “other” balanced translocation product) occurred as a very early mitotic event in an initially normal diploid zygote. More specifically, this may have occurred at least before differentiation of trophoblast and inner cell mass in the 16-cell stage. To explain the phenotypic variability at the tissue level, we speculated that the malformations of lungs, diaphragm and kidneys would associate with a much higher level of mosaicism than the normally developed tissues of heart and liver. This would thus mirror a situation in which the post zygotic mutation arose in an important lung-and diaphragm specific precursor cell population and time window. However, such a linear correlation between level of mosaicism and tissue pathophysiology could not be confirmed in our case. Associations between the severity of the (whole) clinical phenotype and the percentage of abnormal cells are generally known for the most common autosomal aneuploidy (trisomy 21), yet the mechanism by which this aneuploidy results in the complex heterogeneous phenotype cannot be attributed to mosaicism alone and is therefore still under discussion [43]–[44]. Differences in default gene-expression profile requirements between tissues, and subsequently variable susceptibility for loss or gain of different chromosome areas could account for the tissue specific pathology in multi-system diseases in general and in mosaic states as well.

Finally, uncultured tissues are in general better able to reveal true chromosomal mosaicism frequencies as compared to cultured tissues, since culturing may introduce a selection bias that distorts the percentages of abnormal cells. However we were not able to detect the low-mosaic der (5) in uncultured AF-cells using MLPA, yet did detect a high-mosaic der(5) in cultured AF cells. From this it can be concluded that the level of mosaicism in uncultured cells was probably too low to be detected with MLPA. These kind of discrepancies have been described in some areas of tissue-specific mosaicism, like mosaic trisomy 20 [45]. However, in the present case there were no clues at all for tissue limited mosaicism as explained above.

### Mosaic unbalanced translocation (5;12) and CDH

Chromosomal abnormalities are detected in 9.5%–34% of prenatally diagnosed CDH cases [46]. The most common identified aneuploidy is trisomy 18 and smaller structural rearrangements (microdeletions/duplications) of the distal arms of chromosome 1q, 8p and 15q are relatively common in complex-CDH subjects as well. However, structural abnormalities of almost all chromosomes in association with diaphragm defects have been described in literature, including those of the short arms of chromosomes 5 and 12 [London medical Databases version 1.0.19]. Unfortunately, the candidate genes responsible for malformation of the diaphragm remain to be determined for both these regions, yet literature suggests that the candidate region for diaphragm defects on 5p most likely overlaps with the CdLS critical region only. In 2004 Krantz et al. [33] described a child with classical symptoms of CdLS and a balanced, de novo t(5;13)(p13.1;q12.1) thereby disrupting the NIPBL gene. However, this child did not show a diaphragm defect. In contrast, Hulinksy et al [31] did identify a child with the CdLS phenotype, CDH and a pure interstitial deletion of 5p (del(5)(p13.1;p14.2)). Whether NIPBL is indeed the causative gene for CdLS associated diaphragm defects needs to be determined. In our own CDH cohort we have 1 patient with both the CdLS-phenotype and a mutation in exon 9 of the NIPBL gene. However, since CDH is only rarely associated with CdLS (5%) and routine NIPBL mutation screening is only recently been put into standard practice, more specific cases featuring both need to be determined. It is also possible, that a combination of CDH and CdLS is often lethal and therefore rarely notified. Low-mosaicism in our case may have caused changes in the (severity) of the CdLS phenotype as repeatedly suggested in literature.

We also compared our patients remaining characteristics to the features described in literature for both genomic areas. Based on this comparison half of the features presented in our patient could be attributed to both chromosomal areas, while some were more associated with the 5p region. Hulinksy et al. [31] suggested that some of the atypical CdLS characteristics in their patient (hypoplastic kidneys, adrenal gland abnormalities and multipele cysts) are caused by haploinsufficiency of other 5p13.1- p14.2 genes. However, retrospective analysis of our patients' MRI did not show evidence for cysts and adrenal gland defects. Moreover, renal abnormalities (primarily vesicouretral reflux and cysts) are described for CdLS after all. Since our case is not a pure monosomy and very early prenatal dysmorphology hampers identification of some clinical features, direct comparison between this literature case and our patient remains troublesome.

Focusing on CDH and genetic mosaicism: the earlier mentioned Pallister-Killian syndrome is the most common CDH-associated syndrome caused by tissue-limited mosaicism. In a small study more than ten years ago, Donnenfeld et al [47] tried to evaluate the overall percentage of tissue-specific mosaicism among fetuses with prenatally diagnosed diaphragmatic hernia and found in one out of 13 fetuses a mosaic isochromosome 12p. However, the diagnostic techniques used in this study were limited and the number of investigated patients low. Only a few other case-reports on CDH and genetic mosaicism have been published since, featuring the chromosomes Y and 16. None have described a mosaic der 5 t(5;12).

### Mosaicism and detection with high–resolution SNP array

In general it is very difficult to estimate the true incidence and significance of genetic mosaicism in human congenital anomalies. The application of high-resolution SNP arrays allows for an easier and more sensitive detection of this type of genetic aberrations for several reasons. First, SNP arrays are shown to be able to detect levels of mosaicism as low as 5% using the B-allele frequency as a more sensitive measurement compared to the more difficult detection of subtle down- or up crease of log R ratios (which only can be used in Oligo based arrays). Second, the possible use of low amounts of DNA from any kind of (uncultured) tissue diminishes the technical failures due to the in-vitro cell culturing process [29]. So, the wide application of these methodologies in diagnostic and research settings will increase our knowledge and ability to diagnose patients with chromosomal mosaicism and allow for better genotype-phenotype correlations.

## Supporting Information

Table S1 **Molecular-Cytogenetic results of the mosaic unbalanced translocation (5;12) in several affected and un-affected tissues.**
(XLS)

Table S2 **Phenotypic comparison of our patients' characteristics with clinical info of the involved chromosomal regions known from literature.**
(XLS)
